# Incomplete Spoiling Correction for 3D $T_{1}$ Mapping

**DOI:** 10.1002/mrm.70559

**Published:** 2026-08-12

**Authors:** Gabriela Belsley, Damian J. Tyler, Matthew D. Robson, Elizabeth M. Tunnicliffe

**Affiliations:** ^1^ Oxford Centre for Clinical Magnetic Resonance Research, Radcliffe Department of Medicine University of Oxford Oxford UK; ^2^ Perspectum Oxford UK

**Keywords:** 3D $T_{1}$ mapping, extended‐phase‐graphs, incomplete spoiling, steady‐state

## Abstract

**Purpose:**

Assess the impact of incomplete spoiling and spatial saturation on $T_{1}$ mapping using the variable flip angle (VFA) spoiled gradient recalled echo sequence (SPGR). Develop a correction for incomplete spoiling that results in an accurate estimate of $T_{1}$, essential for quantitative MRI applications.

**Methods:**

A correction factor, derived from extended phase graph simulations, was applied to the measured SPGR signal to account for deviations from the theoretical steady‐state signal. An iterative fitting process was used, refining the incomplete spoiling correction factor at each step based on the updated $T_{1}$ estimate. The incomplete spoiling correction method was validated against inversion recovery spin echo in phantom experiments and applied to in vivo data at 3T. Additionally, the hypothesis that spatial saturation disrupts the steady‐state was also tested and validated in a phantom.

**Results:**

Without a correction, the median $T_{1}$ error was +7.4% (IQR [4.4, 9.6]%) in both simulations and phantom experiments. After applying the correction, the median $T_{1}$ error decreased to −0.3% (IQR [−2.5, 2.1]%). In vivo, incomplete spoiling caused $T_{1}$ overestimation of 10–50 ms depending on $B_{1}^{+}$ inhomogeneity. Spatial saturation induced on average a −10% $T_{1}$ bias.

**Conclusion:**

A correction for incomplete spoiling is essential to achieve an accurate measure of $T_{1}$ using the VFA SPGR method, addressing systematic $T_{1}$ overestimation when using this $T_{1}$ mapping technique. Furthermore, other mechanisms that may disrupt the steady‐state signal, such as spatial saturation, should be considered.

## 1 Introduction

$T_{1}$ mapping has been applied to multiple pathologies including the assessment of liver fibrosis and stratification of patients with metabolic dysfunction associated steatotic liver disease (MASLD) [1, 2] and steatohepatitis (MASH) [3], among aetiologies [4, 5, 6, 7]. Whole‐liver $T_{1}$ mapping is enabled through the variable flip angle (VFA) spoiled gradient recalled echo (SPGR) acquisition [8]. VFA $T_{1}$ mapping is used in contrast‐enhanced MRI to assess cell viability and liver function [9]. VFA $T_{1}$ mapping of fat has also been proposed both as a biomarker of tissue oxygenation [10] and as a means of correcting $T_{1}$‐weighting bias in chemical‐shift‐encoded MRI performed with high flip angles for proton density fat fraction quantification [11].

The VFA SPGR method uses multiple RF pulses to drive the longitudinal magnetization to a steady‐state. However, any deviation of the SPGR signal from the predicted steady‐state signal will result in inaccurate $T_{1}$ values when fitting the signal to the steady‐state model. To achieve short acquisition times and a strong $T_{1}$‐weighted contrast, the SPGR sequence is run with a TR that is short compared to $T_{2}$. For SPGR, the steady‐state model assumes that residual transverse magnetization is fully dephased before the next RF excitation pulse. Otherwise, the signal will present $T_{2}$ contrast and thus deviate from the steady‐state signal, which presumes only longitudinal magnetization is affected by the RF pulses. In the liver, assuming a normal iron concentration of 1 mg Fe*/*g (dry weight of tissue), the $T_{2}$ is approximately 22 ms [12]. For a TR of 4.1 ms only 17% of the transverse magnetization will undergo $T_{2}$ decay before the next RF excitation pulse, highlighting the need to spoil the transverse magnetization.

RF spoiling applies pulses with incrementing phase at each TR, causing the spins to acquire distinct phase evolutions and cancel each other's contribution through destructive interference. Zur et al. [13] showed that a phase increment of 117° or 123° achieves nearly complete spoiling for a wide range of physiologically relevant $T_{1}$ s/TR, $T_{2}$ s/TR, and excitation FAs between 0° and 90°. Figure 1a highlights the importance of carefully choosing the RF phase increment: even for a 117° phase increment at an excitation FA of 15°, the SPGR signal—simulated through the extended‐phase‐graph (EPG) framework—deviates from the steady‐state. An RF phase increment of 50°, default setting on some clinical scanners, results in a signal lower than the steady–state achieved from a perfectly spoiled acquisition (Figure 1b). The lower signal mimics a longer $T_{1}$ resulting in $T_{1}$ overestimation.

**FIGURE 1:**
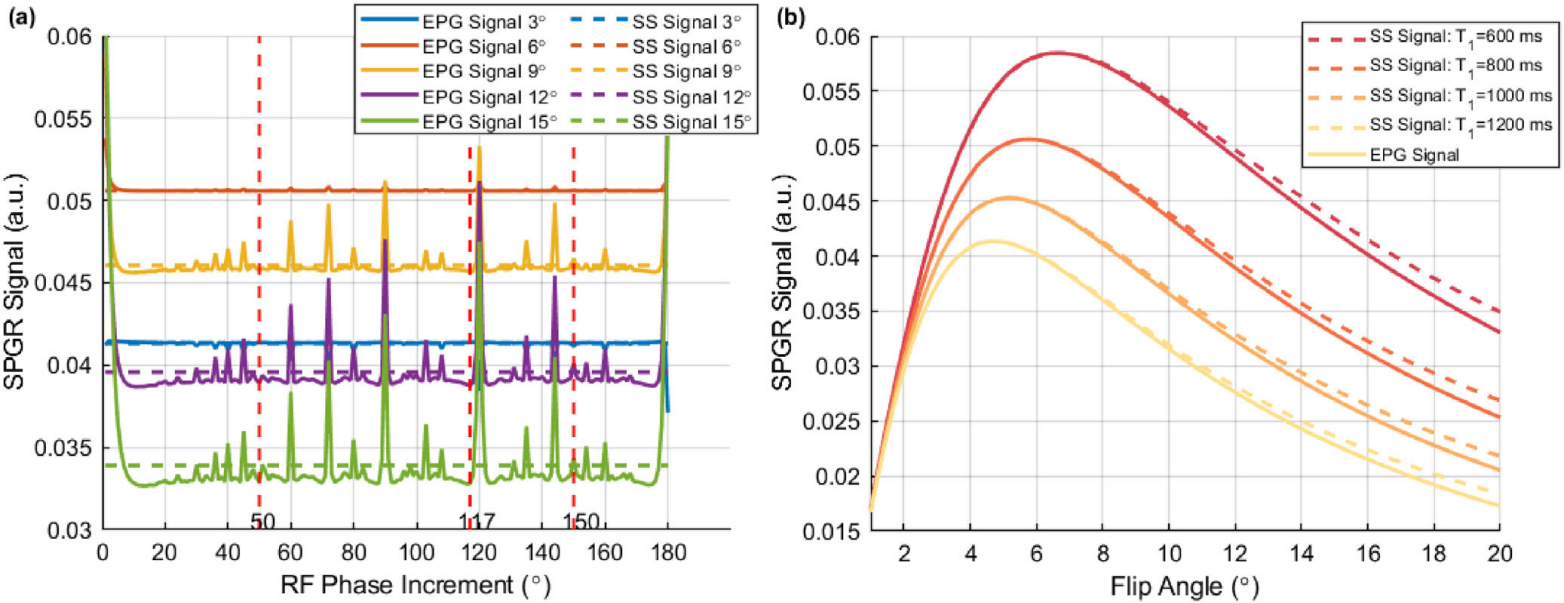
(a) SPGR signal dependence on RF spoiling phase increment simulated for FAs of 3° (blue), 6° (orange), 9° (yellow), 12° (purple) and 15° (green) through the extended‐phase‐graph (EPG) framework (solid line) and the steady‐state (SS) theoretical equation (dashed line) with parameters: $T_{1}$ = 800 ms, $T_{2}$ = 30 ms, TR = 4.1 ms and 1500 RF pulses. A careful choice of the RF phase increment is crucial to avoid large deviations of the signal from the theoretical steady‐state prediction. The RF spoiling phase increments chosen by the different vendors are highlighted by the vertical red dashed lines: 50° for Siemens, 117° for GE and 150° for Philips. (b) SPGR steady‐state signal as a function of the excitation FA represented in dashed lines for different $T_{1}$ values of 600 ms (red), 800 ms (orange), 1000 ms (light orange), and 1200 ms (yellow). Solid lines represent the true signal, with matching $T_{1}$ colors, calculated through EPG simulations which model the effect of incomplete spoiling. The simulations used a TR = 4.1 ms, $T_{2}$ = 30 ms and an RF phase increment of 50°. For these parameters, incomplete spoiling decreases the SPGR signal compared to the steady state signal, and the decrease is more severe as the FA value increases above the Ernst Angle.

Spatial pre‐saturation pulses can be used to suppress the signal from tissues outside of the field of view (FoV) [14]. Its main application is to reduce flow, motion, and aliasing artifacts along the PE direction, arising from tissue excited outside of the FoV. Spatial saturation pulses should not interfere with the ability of the magnetization to reach steady–state. One common approach interleaves two saturation pulses within each phase‐encode acquisition block. We hypothesized this implementation perturbs the steady–state, biasing $T_{1}$ estimates.

The aim of this study was to develop a correction for incomplete spoiling in the VFA SPGR sequence to achieve accurate $T_{1}$ estimates of the liver. The correction is based on EPG simulations across physiological liver TR/$T_{1}$ and TR/$T_{2}$ ranges. We also assessed spatial saturation effects on T_1_ accuracy.

## 2 Methods

### 2.1 Algorithm for Incomplete Spoiling

The six key factors that affect incomplete spoiling of the magnetization are the excitation FAs, the expected $T_{1}$ and $T_{2}$ range of the tissue of interest, the sequence TR, and the RF phase increment and a gradient spoiling, which can introduce diffusion effects when dephasing is much greater than the minimum of 2pi per voxel. For a phase increment of 50° we simulated transverse signals using EPG [15, 16] for $T_{1}$ = 500–1600 ms (100 ms steps), $T_{2}$ = 20–35 ms (5 ms steps) [17], and FA = 1°–20° (1° steps). A fixed TR of 4.1 ms was used as this allows whole liver coverage within a 15 s breath‐hold with parallel imaging. The k‐space center was reached after 6.9 s (1683 TRs), exceeding the 3*$T_{1}$ requirement to achieve steady–state for a perfectly spoiled sequence (4.8 s for tissue $T_{1}$ = 1600 ms).

The incomplete spoiling correction transformed the acquired signal to the steady‐state space. The acquired signal was multiplied by the ratio between the theoretical steady‐state signal (Equation 1a) and the simulated EPG signal at the estimated values of $T_{1}$ and true FA (Equation 1b).

(1a) $$S_{\text{steady}-\text{state}}=M_{0}\frac{1-\exp\left(\frac{-\mathrm{TR}}{T_{1}}\right)}{1-\cos\left(\alpha \right)\exp\left(\frac{-\mathrm{TR}}{T_{1}}\right)}\sin\left(\alpha \right),$$

(1b) $$S_{\text{Corrected}}=S_{\text{acquired}}\cdot \frac{S_{\text{steady}-\text{state}}}{S_{\mathrm{EPG}}},$$

where α is the excitation FA, $M_{0}$ is a numerical constant including the proton density, signal decay due to $T_{2}^{⋆}$ relaxation and the $B_{1}^{-}$ receive sensitivity; TR is the repetition time and $T_{1}$ is the longitudinal relaxation time.

The correction was incorporated into the non‐linear least squares (NLLS) $T_{1}$ fitting procedure following an iterative approach. An initial estimate of $T_{1}$ was obtained from the two lowest distinct flip angles (i.e., the two lowest non‐equal flip angles), by computing the ratio ρ = S₁/S_2_ of the two SPGR signals, after correcting the nominal flip angles using the B1^+^ map, and solving the steady‐state SPGR equation analytically for E₁ = (sin α₁ − ρ sin α_2_)/(sin α₁ cos α_2_ − ρ sin α_2_ cos α₁), from which $T_{1}$ = −TR/ln(E₁). The lowest flip angles were selected because spoiling‐related $T_{1}$ errors are amplified at larger flip angles (Figure 1b). For each voxel, the nominal FAs were first corrected using the $B_{1}^{+}$ map to obtain the true FAs. The initial $T_{1}$ estimate and the $B_{1}^{+}$ corrected FAs were used to interpolate the incomplete spoiling correction factor curves (Figure S1), calculated offline for a grid of $T_{1}s$, FAs and $T_{2}$ of 30 ms. The acquired SPGR signal was multiplied by the interpolated correction factor and subsequently fitted to the theoretical steady‐state model to refine the initial $T_{1}$ estimate using a NLLS fit. The incomplete spoiling correction was iterated at each step with the updated $T_{1}$ estimate (Figure S2). Iteration stopped when consecutive T₁ estimates differed by less than 1 ms, or after a maximum of five iterations. In practice, convergence was typically reached after 2–3 iterations for both phantom and in vivo dataset.

Because liver $T_{2}$ was not measured with the VFA SPGR sequence, it was necessary to use a fixed $T_{2}$ value that would give the best overall results for the $B_{1}^{+}$ factor, $T_{1}$ and $T_{2}$ parameter space observed in the liver. For a liver $T_{2}$ range of 20–35 ms [17] (at 3T), a $T_{2}$ of 30 ms gave the smallest error between the true $T_{1}$ and the fitted $T_{1}$ including our spoiling correction. Errors remained < 2.3% across the full range of T_2_s (20–35 ms) [17], $T_{1}$ s (700–1200 ms) [18] and $B_{1}^{+}$ factors (0.59–1.14) [19]. The nominal FA set used was [2, 2, 15, 15]°.

### 2.2 Simulations

Monte Carlo simulations compared $T_{1}$ error with and without incomplete spoiling correction. The error was simulated over a range of $T_{2}$ s (20–35 ms in steps of 5 ms) and $T_{1}$ s (500–1600 ms in steps of 100 ms), with 10 000 repetitions per $T_{1}$ and $T_{2}$ combination. The EPG and the steady–state simulated signals were corrupted with zero mean additive Gaussian noise, corresponding to an SNR of 25 at a nominal FA of 2°, $T_{1}$ of 800 ms and TR of 4.1 ms. We used a maximum B_1_^+^ = 1.14 [19] since incomplete spoiling error increases with flip angle.

### 2.3 Spatial Saturation

The total duration of a single breath‐hold SPGR acquisition with parallel imaging per FA was 15 s. The acquisition time with parallel imaging is sufficient for liver tissue to reach steady–state. Thus, no difference is expected in the $T_{1}$ maps from using parallel imaging. However, we hypothesized that the $T_{1}$ difference observed with parallel imaging on and off originated from the location of the saturation pulses in the SPGR sequence. To test this hypothesis, phantom data was acquired for the SPGR sequence with four different combinations of parallel imaging and saturation, named special saturation on the Siemens' parameter card and applied in the foot‐head direction:

1. Saturation On and Caipirinha On.

2. Saturation On and Caipirinha Off.

3. Saturation Off and Caipirinha On.

4. Saturation Off and Caipirinha Off.

### 2.4 Phantom and In Vivo Scans

Imaging data were acquired on phantoms and 10 healthy volunteers on a 3T Prisma scanner (Siemens Healthineers, Forchheim, Germany) with software version *syngo MR E11*, using a spine array coil and an 18‐channel array coil. Each volunteer underwent three repeated VFA SPGR $T_{1}$ acquisitions, with $B_{1}^{+}$ and $B_{0}$ maps acquired for each repeat and applied to the $T_{1}$ maps. Volunteers were scanned according to our institution's ethical practices and gave informed consent.

Two different phantoms were supplied by Perspectum Ltd. (Oxford, UK) and imaged: marked as *QC0025* and *FDA4‐T1*. The *QC0025* phantom contained 15 vials: 5 agar gel vials doped with NiCl_2_ with $T_{1}$ s (at 3T) between 548 and 1429 ms which were used in this work; 5 vials had varying $T_{2}$* values, and the last 5 vials had varying fat fractions. This phantom was used for the spatial saturation experiments. The *FDA4‐T1* phantom contained 14 vials each of which contained 3% agar gel with NiCl_2_ as the doping agent. This resulted in vials with $T_{1}$ s (at 3T) ranging from 367 to 1699 ms in steps of approximately 100 ms, an average $T_{2}$ of 45 ms and no fat.

All sequences used for acquisition were unmodified product sequences that are widely available on any scanner independent of manufacturer, thereby facilitating reproducibility and widespread use of the method in clinical studies.

$T_{1}$ mapping used a 3D VFA SPGR with Dixon [20] fat‐water separation. Water‐only images were used for calculating the $T_{1}$ maps. Acquisition parameters: FoV = $450\times 366\times 3\;mm^{3}$, matrix = 320×260×48, TR/TEs = 4.1/[1.23/2.46] ms, Bandwidth (BW) = 1040 Hz/pixel, CAIPIRINHA [21] with 3× acceleration along the slice direction and 24 separate GRE reference lines. Nominal in vivo FAs were [$2^{\circ }$, $2^{\circ }$, $15^{\circ },$ $15^{\circ }]$, each acquired during a 15 s end‐expiratory breath‐hold. This FA set was selected based on a previously published optimization framework [22], which minimizes $T_{1}$ variance by accounting for noise propagation from both the SPGR images and the $B_{1}^{+}$ map across a range of $B_{1}^{+}$ factors and $T_{1}$ s values observed in the liver at 3T, yielding an optimal set of [2°, 3°, 15°, 15°]. The low FA was modified from 3° to 2° to additionally enable in vivo SNR estimation, obtained by subtracting two identically acquired images. The signals acquired at all nominal FAs, including repeated acquisitions at the same FA, were used directly in the voxel‐wise liver $T_{1}$ estimation pipeline depicted in Figure S2, without prior averaging, to avoid propagating potential respiratory misalignments into an averaged image. For the phantom experiment, additional low FAs were acquired to investigate incomplete spoiling effects on FA values, resulting in a total FA set of [$2^{\circ }$, $2^{\circ }$, $3^{\circ },4^{\circ },15^{\circ },$ $15^{\circ }]$.

$B_{1}^{+}$ mapping used a 2D multi‐slice GRE EPI sequence with fat saturation and nominal FAs of $65^{\circ }$ and $130^{\circ }$ [22], each a 10 s breath‐hold. $B_{1}^{+}$ maps included corrections for slice profile effects and $B_{0}$‐gradient through‐slice variations [23]. $B_{0}$ mapping acquired through a 2D multi‐slice double echo spoiled GRE, were used for distortion correction of the GRE‐EPI images and modeling of $B_{0}$‐variations through slice in the $B_{1}^{+}$ map [20]. Acquisition parameters for the $B_{1}^{+}$ and $B_{0}$ mapping are described in the Supporting Information.

The gold standard (GS) $T_{1}$ map for the phantoms consisted of a slice‐selective inversion recovery spin echo (IR SE) acquisition with 15 inversion times from 25 to 5000 ms (Figure S9).

Fifteen masks were manually drawn over the liver on the high‐resolution SPGR acquisition (Figure S3), acquired at a FA of 15°, for the slices matching the location of each of the 15 slices in the GRE‐EPI acquisition. The mask was down‐sampled to the GRE acquisitions for calculation of the $B_{1}^{+}$ and $B_{0}$ maps. Subsequently, the masked $B_{1}^{+}$ map was interpolated along the three dimensions to the same spatial resolution as the SPGR acquisition, such that the interpolated $B_{1}^{+}$ factor was used to correct the SPGR nominal FAs at a voxel level. Each $T_{1}$ map was calculated for the masked liver region, using the processing pipeline depicted in Figure S2, resulting in 30 $T_{1}$ maps.

Vessels were removed from the liver‐masked $T_{1}$ map by masking out pixels with $T_{1}$ > 1150 ms and $B_{1}^{+}$ < 0.3. Pixel‐wise difference between vessel‐ and liver‐masked $T_{1}$ maps with and without the incomplete spoiling correction were calculated for the central slice in the liver. For each volunteer, the mean and standard deviation of these differences were computed across all pixels and the three repetitions.

## 3 Results

### 3.1 Simulations

Without incomplete spoiling correction, the relative $T_{1}$ error reached 7.7% at $T_{1}$ = 1200 ms and $T_{2}$ = 35 ms (Figure 2a). After applying the correction, the relative $T_{1}$ error was approximately zero at $T_{2}$ = 30 ms, as this was the fixed $T_{2}$ adopted for the correction, and reached −2.1% at $T_{2}$ = 20 ms and 1.3% at $T_{2}$ = 35 ms.

**FIGURE 2:**
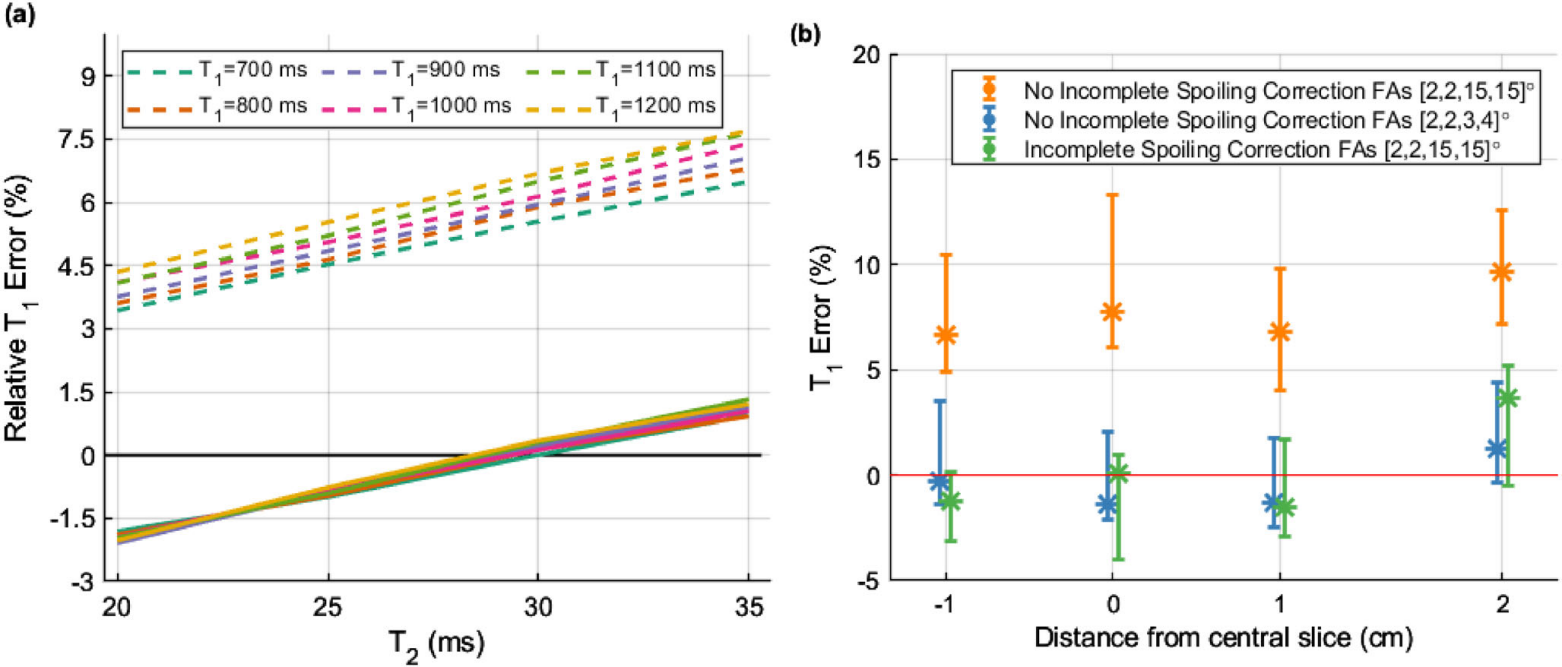
(a) Simulated relative error in water $T_{1}$ (%) due to incomplete spoiling (dashed lines) for a range of true $T_{1}$ s between 700 and 1200 ms and a range of $T_{2}$ s between 20 and 35 ms. The water $T_{1}$ is always overestimated. The relative water $T_{1}$ error decreases after applying the incomplete spoiling correction (solid lines). These simulations used an RF phase increment of 50°, TR of 4.1 ms and a FA set of [2, 2, 15, 15]°. (b) Water $T_{1}$ error in percent, relative to the IR SE, without any incomplete spoiling correction applied to the SPGR signals acquired in the *FDA4‐T1 phantom* at FAs of [2, 2, 15, 15]° (orange). The median water $T_{1}$ error was 7.4% with an interquartile range (IQR) of [4.4, 9.6]%. Using the SPGR signal acquired at FAs of [2, 2, 3, 4]° (blue) without incomplete spoiling correction the median water $T_{1}$ error decreased to −0.7% and the IQR to [−3.8, 0.8]%. Applying the incomplete spoiling correction to the signal acquired at FAs of [2, 2, 15, 15]° (green), the median water $T_{1}$ error was −0.3% with an IQR of [−2.5, 2.1]%.

### 3.2 Phantom

Figure 2b shows that the water $T_{1}$ error in the *FDA4‐T1* phantom without incomplete spoiling correction using FAs of [2, 2, 15, 15]° reached a median value of 7.4% with an IQR of [4.4, 9.6]% across all vials for the four central slices. This bias is also observed in the analysis of the residuals of the NLLS fit in the Supporting Information (Figures S4, S5). With correction, the median water $T_{1}$ error decreased to −0.3% (IQR of [−2.5, 2.1]%). Using FAs below the Ernst angle of [2, 2, 3, 4]° resulted in unbiased $T_{1}$ estimates (−0.7% error) without applying the incomplete spoiling correction. The $T_{1}$ precision was not affected by the low FAs as the phantom is a system with a high SNR of 103, (measured from the difference between two repeated 2° acquisitions). The water SPGR $T_{1}$ values were compared against the gold standard IR SE $T_{1}$ values measured in the phantom (Figure S9).

Figure 3 confirms that parallel imaging does not affect the SPGR signal when special saturation is turned off in the *QC0025 phantom*. On the other hand, with saturation on, differences in SPGR signal exceeding 8% and water $T_{1}$ differences of on average 10% were observed between parallel imaging on and off. Without saturation, water $T_{1}$ values agreed regardless of parallel imaging, with smaller fit residuals (Figures S7, S8).

**FIGURE 3:**
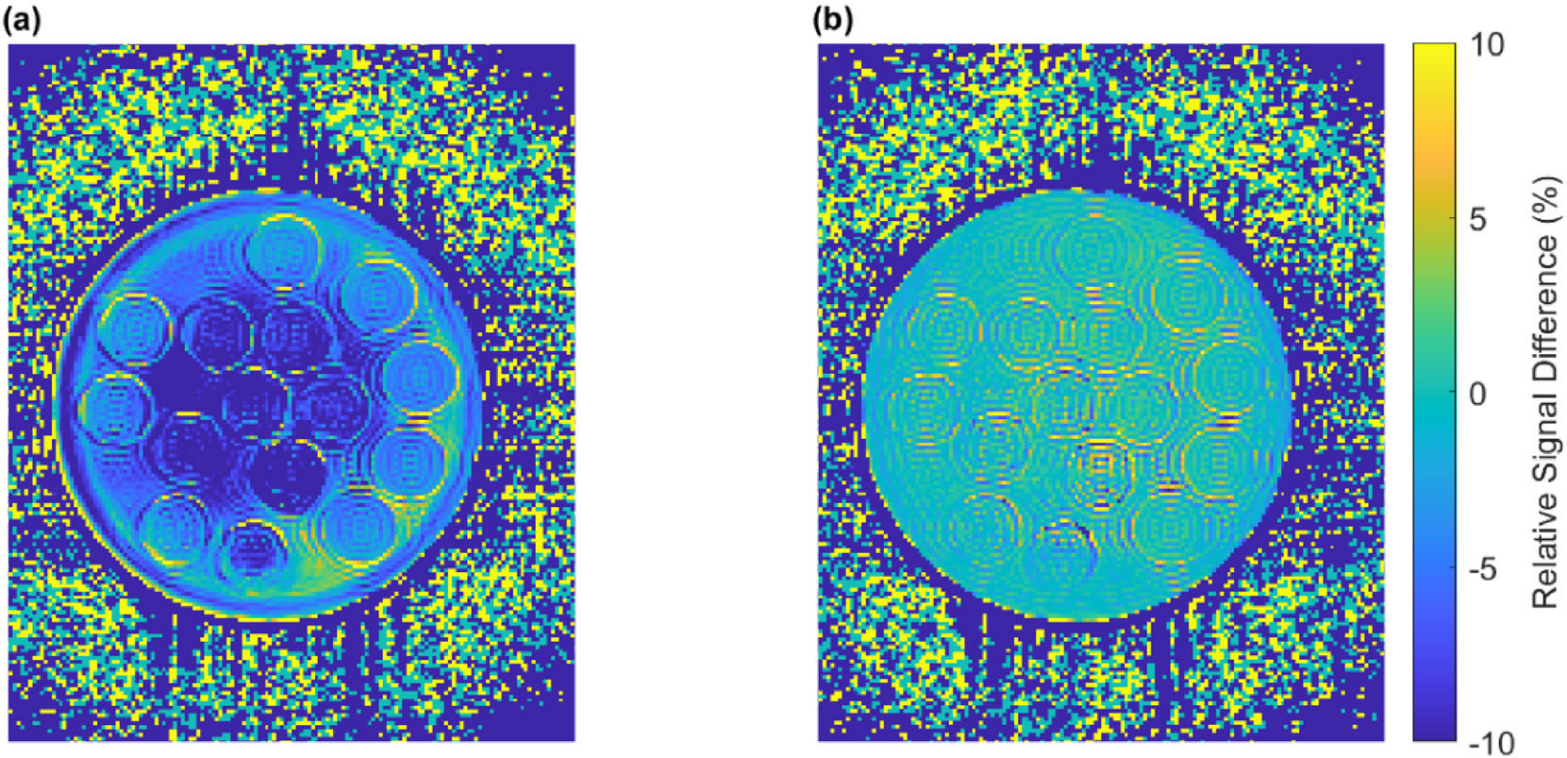
Percent difference in SPGR signals in the QC0025 phantom between parallel Imaging on and off with spatial saturation turned (a) on and (b) off, normalized by parallel imaging off to obtain a relative signal difference. With spatial saturation, parallel imaging resulted in a signal lower by 8% or more relative to parallel imaging off for the central vials which contain $T_{1}$ s varying between 548, 633, 790, 1044, and 1429 ms. Turning saturation off (b) results in very small SPGR signal differences between parallel imaging on and off as expected.

### 3.3 In Vivo

The water‐liver $T_{1}$ map in vivo uncorrected for incomplete spoiling for volunteer 10 was on average 51 ms higher than the corrected map, for the posterior part of the liver (lower part of the liver in Figure 4). The anterior portion of the liver had a smaller average difference of 25 ms as this was a location with a $B_{1}^{+}$ factor less than 1. The posterior part had $B_{1}^{+}$ factors slightly larger than one, resulting in higher excitation FAs and thus more incomplete spoiling.

**FIGURE 4:**
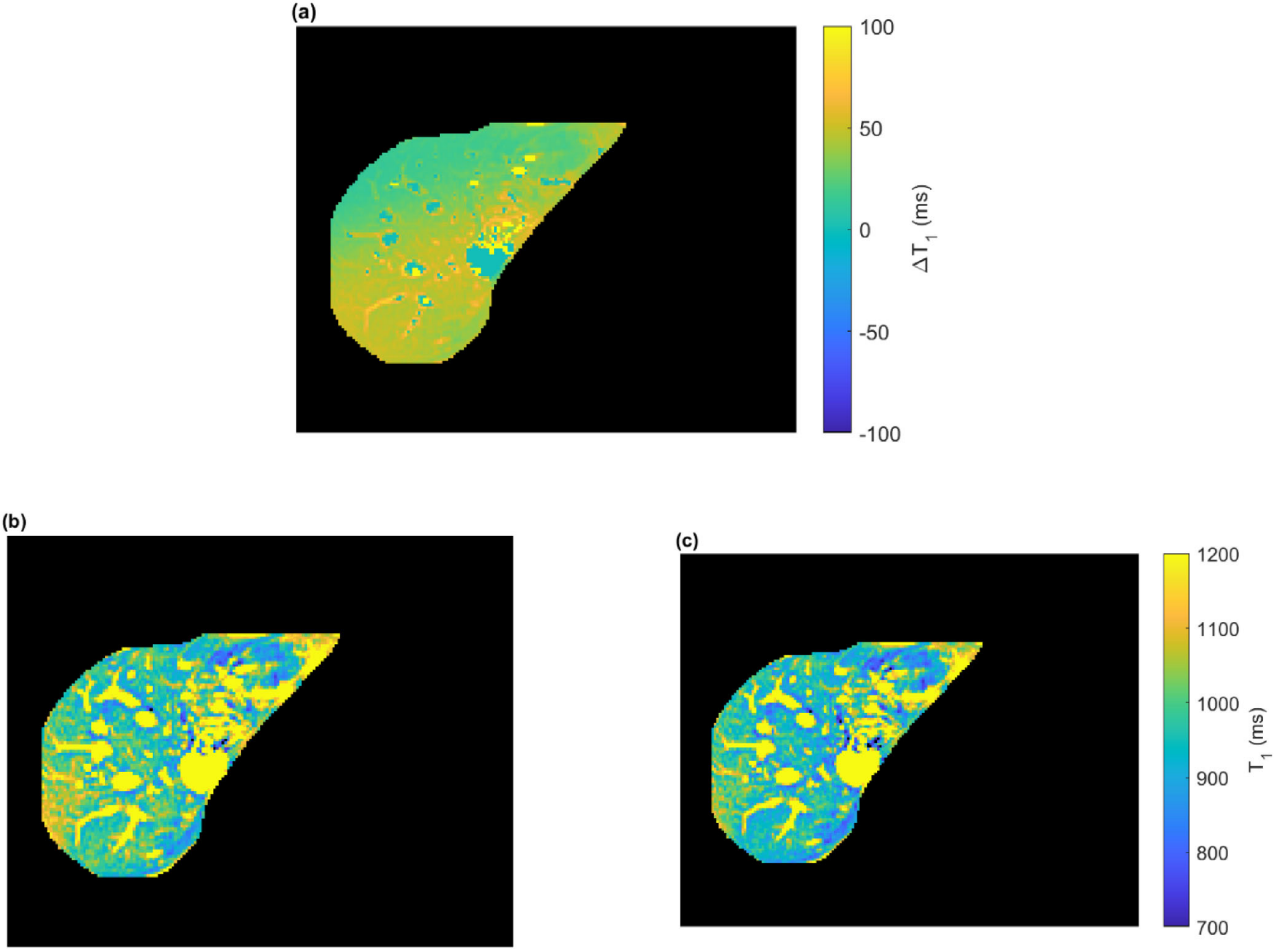
(a) $T_{1}$ map difference between the (b) water $T_{1}$ map uncorrected for incomplete spoiling and the (c) water $T_{1}$ map with the incomplete spoiling correction. The water $T_{1}$ maps were reconstructed from the water‐only SPGR signal acquired at nominal FAs of [2, 2, 15, 15]°. The vertical dimension corresponds to the anterior–posterior direction.

On average across the 10 volunteers, the water‐liver $T_{1}$ is 28 ms higher without correcting for incomplete spoiling (Table 1). In individuals with a higher mean water‐liver $T_{1}$ or in locations with $B_{1}^{+}$ factors above 1, the incomplete spoiling bias results in a $T_{1}$ 50 ms or higher than the true $T_{1}$.

**TABLE 1:** Difference in $T_{1}$ between water‐liver $T_{1}$ maps calculated without and with a correction for incomplete spoiling for the central slice after masking out the vessels.

Volunteer	$∆T_{1}$ μ±σ (ms)	$\mu_{T_{1}}$ not corrected (ms)	$\mu_{T_{1}}$ corrected (ms)
1	29 ± 11	862	834
2	16 ± 7	881	864
3	36 ± 12	1066	1029
4	35 ± 12	991	956
5	36 ± 11	992	956
6	20 ± 10	817	797
7	30 ± 13	855	825
8	22 ± 15	916	894
9	21 ± 12	802	781
10	35 ± 14	975	940

*Note:* The values reported in Column 1 correspond to the mean and standard deviation of the $T_{1}$ differences across the three repetitions for each volunteer. The mean water‐liver $T_{1}$ map values across the three repetitions for each volunteer, using the same vessel mask, is shown in Column 2 without correcting for incomplete spoiling and in Column 3 with the developed correction.

## 4 Discussion

Correcting for incomplete spoiling in 3D VFA SPGR $T_{1}$ mapping is essential to achieve accurate measurements. Short TR drives unspoiled transverse magnetisation to the steady‐state, introducing $T_{2}$ contrast that invalidates fitting of the acquired signal to the steady‐state model (Equation 1a). Simulations showed $T_{1}$ errors of 7.7% at a $T_{1}$ of 1200 ms and a $T_{2}$ of 35 ms when not correcting for this confounding factor. Overestimating water‐liver $T_{1}$ by almost 100 ms could significantly impair clinical utility by misclassifying healthy liver tissue as diseased or overstating the severity of liver fibrosis. Phantom experiments confirmed the simulation predictions. After applying the correction, the median water‐liver $T_{1}$ error decreased to −0.3% (IQR [−2.5 2.1] %), indicating a high accuracy of the proposed correction. In vivo data show a clear overestimation of water‐liver $T_{1}$ in the presence of incomplete spoiling, being worse for the posterior part of the liver where $B_{1}^{+}$ inhomogeneities are greater than 1. On average, incomplete spoiling introduced a positive water‐liver $T_{1}$ bias of 28 ms across the 10 volunteers; however, individual water‐liver $T_{1}$ biases as high as 60 ms were observed in the absence of correction. Depending on its implementation, spatial saturation can bias SPGR water‐liver $T_{1}$ mapping.

Preibisch and Deichmann [24] developed a correction for incomplete spoiling based on an assumed linear relationship between the true $T_{1}$ and the $T_{1}$ predicted by fitting the incomplete spoiling signal to the steady‐state equation. Their correction parameters varied quadratically with $B_{1}^{+}$ (0.7–1.3) for FAs of 4° and 18°, TR = 7.6 ms, and $T_{1}$ = 700–1800 ms. In phantom, their correction reduced $T_{1}$ error from 12% to less than 1%. However, in vivo $T_{1}$ remained 100 ms above IR EPI reference values. It should be noted that their correction is based on fitting $T_{1}$ to a linear form of the steady‐state equation obtained using the small‐angle approximation. This linear approximation has been shown to cause $T_{1}$ inaccuracies [25].

An alternative correction for incomplete spoiling was proposed by Baudrexel et al. [26]. These authors assume the steady‐state signal is valid in the case of incomplete RF spoiling, provided an extra correction is applied to the FA, in addition to the $B_{1}^{+}$ mapping correction. Using an RF phase increment of 50°, they observed systematic errors in $T_{1}$ as high as 13% in phantom and 24.2% in vivo for large FAs of 32°. After applying their proposed incomplete spoiling correction, the $T_{1}$ errors decreased to 0.3% for the phantom, and in vivo to 5.2% for WM, and 9.9% for GM. The approach of Baudrexel et al. [26] aligns with the observation that larger excitation FAs will need larger incomplete spoiling correction factors as a larger amount of longitudinal magnetisation is flipped to the transverse plane. However, their $T_{1}$‐independent correction does not agree with Figure 1b where the deviation between the steady‐state and the incompletely spoiled signal changes as a function of $T_{1}$.

Our work does not make any underlying assumptions regarding the correction's dependence on $T_{1}$ values, nor does it use either the small angle approximation or the linear form of the steady‐state equation. The correction is based on EPG simulations over a range of liver physiological TR/$T_{1}$ and TR/$T_{2}$ values. EPG gives an accurate estimate of the echo intensity in the presence of incomplete spoiling [12], enabling signal rescaling to steady‐state.

The 3D SPGR product sequence used in this work uses a combination of gradient spoiling and RF spoiling with a RF phase increment of 50 degrees. The gradient spoiling lies in the weak spoiling regime with a total dephasing of approximately 7.26 rad (slightly above 2 π) per voxel in the readout direction, with additional spoiling along the slice direction. Both the gradient and RF spoiling are taken into account during the EPG simulations. As shown in our simulations (Figure S6) and consistent with the literature, using larger spoiler gradients—above 280 mT*ms/m [27] or 6 π dephasing per voxel [28]—reduces residual transverse magnetization and the dependence of $T_{1}$ on the RF phase increment. However, as demonstrated in both references [27, 28] and our own simulations, increasing the spoiler gradient area does not eliminate the need for post hoc correction. Importantly, large spoiler gradients are sensitive to diffusion [27, 28] which should be included in the EPG simulations. For our acquisition, diffusion effects are negligible since we are in a weak spoiling regime and the rms diffusion displacement is small compared to the voxel size ($\sqrt{\left\langle x^{2}\right\rangle }$ ≈ 3 μm per TR, voxel size 1.4 mm) and introduce only 0.016 rad ($\gamma A_{G}\sqrt{\left\langle x^{2}\right\rangle }$) of additional dephasing. Figure S6 shows a minor difference in the $T_{1}$ estimate when diffusion effects are included and the gradient spoiler is close to 2 π. In practice, the large spoiler gradient moments required for the strong spoiling regime would increase TR and preclude breath‐hold scanning. Furthermore, the short TR is also constrained by the requirement for two echo times for DIXON‐based fat‐water separation, leaving insufficient time within each TR to accommodate larger spoiler gradient waveforms. Corbin et al. [28], also showed that an RF phase increment of 50 degrees is particularly sensitive to $B_{1}^{+}$ inhomogeneities and T2, emphasizing the importance of an accurate $B_{1}^{+}$ map. This sensitivity is accounted for in our work, as the true FAs are used to interpolate the incomplete spoiling correction factor curves.

A careful analysis of the location of the spatial saturation building block within the sequence diagram is essential for determining whether it interferes with the steady‐state SPGR signal. When the saturation pulses are interleaved with the partition acquisition block and the duration of the saturation module is larger than TR, then the longitudinal magnetisation will undergo a larger recovery than the amount flipped by the next RF excitation pulse, violating the steady‐state condition. Our parallel imaging experiments can detect such disruptions when sequence diagrams from vendors are unavailable. In this work we turned off saturation, given the differences in $T_{1}$ between parallel imaging on and off. No flow artifacts were observed, though monitoring remains advisable. In cases where preparation pulses are desired, the steady‐state equation needs to be modified as shown by Wennen et al. [29]. They also showed an underestimation of $T_{1}$ when not accounting for the temporal gap introduced by the saturation pulses. However, note that their analysis does not include corrections for $B_{1}^{+}$ inhomogeneities or incomplete spoiling, which further changes the steady‐state model.

One limitation of our correction method is the use of a fixed $T_{2}$ value for the liver. Simulations suggest that errors remain < 2% for T_2_ values within ±10 ms of the chosen value (30 ms). Baudrexel et al. [26] also used a fixed $T_{2}$ and Preibisch and Deichmann [24] didn't take $T_{2}$ into account but also simulated $T_{1}$ errors of less than 5% for $T_{2}$ s varying between 60 to 110 ms in the brain using an RF phase increment of 50 degrees. Corbin et al. [28] also showed that φ₀ = 50° exhibits higher T2 sensitivity compared to other increments, such that a mismatch of 10 ms in the T2 assumed for the correction can produce residual errors of up to 2.7% at high B1^+^ efficiencies. Nevertheless, when multiparametric methods are used that simultaneously estimate $T_{1}$ and $T_{2}$, the $T_{2}$ values can be incorporated into EPG simulations for improved accuracy.

The 2‐echo Dixon VFA SPGR is appealing for $T_{1}$ mapping in the liver as it allows to calculate fat and water‐only whole‐liver images from a single breath‐hold. However, when using FAs near or above the Ernst Angle, different $T_{1}$‐weightings are introduced in the fat and water signal [30]. This results in an overestimation of the fat‐fraction which consequently results in an underestimation in water‐liver $T_{1}$, and this error increases as the fat fraction increases [8]. We have shown that for fat fractions up to 10% the $T_{1}$ error is 3% [8]. To obtain an accurate water‐liver $T_{1}$ for patients with fat percentages in the liver higher than 10%, the method would require more than two echoes together with a correction for $T_{1}$‐weighting introduced by FAs near or above the Ernst angle [8].

Given the multiple breath‐hold acquisitions required for VFA SPGR (4 FAs), $B_{1}^{+}$ map (2 FAs), $B_{0}$ map (1 FA), slight breath‐hold misalignments between acquisitions were observed in some volunteers. Attempts to register the FAs in the $B_{1}^{+}$ map or the SPGR were undertaken with different registration methods, including groupwise registration, which is considered state‐of‐the‐art for quantitative imaging [31]. However, registration was abandoned as differences in $T_{1}$ of 50 ms or higher, between registered and non‐registered $T_{1}$ maps, were observed for more than half of the pixels in a central liver slice of a volunteer without any discernible breath‐hold misalignment. A hypothesis is that registration shifts anatomy to a location with different $B_{1}^{-}$ factors and both the $B_{1}^{-}$ and the proton density are assumed to remain constant on a pixel‐by‐pixel basis over the various acquisitions used to determine the $B_{1}^{+}$ factor or estimate $T_{1}$. In our repeatability study [8] a small within‐subject standard deviation of 21 ± 5 ms was achieved without registration, suggesting that small misalignments between the different acquisitions do not substantially affect the $T_{1}$ map calculation for the central portion of the liver. The $T_{1}$ maps near the liver edges were more sensitive to misalignments; however, these extremity regions will always be problematic as they correspond to areas of large $B_{0}$ inhomogeneities as well as partial volume effects.

Although this work was carried out in the liver at 3T, the proposed framework could, in principle, be applied to other field strengths and anatomical regions using the VFA SPGR‐based $T_{1}$ mapping. This would require new EPG simulations tailored to the $\mathrm{TR}/T_{1}$ and $\mathrm{TR}/T_{2}$ ranges of the tissue of interest, as well as to the RF spoiling phase increment adopted by the scanner manufacturer. However, the correction algorithm itself and the overall image‐processing pipeline would remain unchanged.

## 5 Conclusion

A correction for incomplete spoiling is crucial for accurately determining $T_{1}$ values using the 3D VFA SPGR sequence. Spatial saturation can also disrupt the steady‐state signal, introducing an additional source of bias in $T_{1}$ measurements. The incomplete spoiling correction proposed leverages EPG simulations across physiological ranges of liver TR/$T_{1}$ and TR/$T_{2}$ values at 3T. This approach is flexible and can be easily adapted to other anatomical regions, field strengths, or RF phase increments.

## Funding

This work was supported by funding from the Engineering and Physical Sciences Research Council (EPSRC), Medical Research Council (MRC) [grant number EP/L016052/1], and the NIHR Oxford Biomedical Research Centre.

## Conflicts of Interest

Gabriela Belsley is an employee and shareholder of Siemens Healthineers. Matthew D. Robson is an employee and shareholder of Perspectum Ltd. The other authors declare no conflicts of interest.

## Supporting information


**Data S1:** Supporting Information.
**Figure S1:** Incomplete spoiling correction factor given by the ratio between the theoretical steady‐state signal and the extended‐phase‐graph signal as a function of the true FA in degrees for a range of T1svarying between 500 ms (blue) and 1600 ms (green). Representative T1values, observed in the liver of healthy volunteers, of 800 ms (orange), 1000 ms (yellow) and 1200 ms (purple) are also plotted. This plot shows the correction factor varies with the true FA as well as T1, hence the iterative procedure for fitting T1.
**Figure S2:** Flow diagram to calculate the T1 map corrected for incomplete spoiling using extended–phase‐graph simulations and corrected for B1+ inhomogeneities using a B1+ map.
**Figure S3:** Liver mask in red drawn on the VFA SPGR high resolution image at FA 15°.
**Figure S4:** (a) T1Map of the FDA4‐T1 phantom without correcting for incomplete spoiling. Red point showing the location of the voxel (126176) used in the analysis of residuals. (b) NLLS fit (dashed blue line) to the acquired data (red stars) without correcting for incomplete spoiling and the corresponding (c) residuals. The fitted T1was 1090‐ms and the sum of the residuals squared (SSR) was 43.0 (arbitrary signal units). The residuals showed a pattern similar to the steady‐state model curve indicating an underlying bias.
**Figure S5:** The fitted T_1_ was 1016ms which is very close to the gold‐standard T_1_ of 1022ms. For an SNR of 157 at a FA of 3°, the noise level was 2.2, and the residuals of the fit to the corrected data were all less than or equal to this noise level.
**Figure S6:** Error in T_1_ for SPGR EPG simulations with varying spoiler gradient areas corresponding to dephasing of 2pi (33.55 mT/m), increasing in steps of 2pi/10 until 10pi (167.75 mT/m) and including diffusion (blue) with diffusion coefficient of 1.1 × 10^−3^ mm^2^/s and excluding diffusion effects (red). TR = 4.1 ms, T_1_ = 800ms, T2 = 30 ms, spoiler gradient duration = 0.5 ms, pixel size 1.4 mm.
**Figure S7:** T_1_ error in percent, relative to the gold standard method inversion recovery spin echo, with saturation (a) on and (b) off. Blue stars represent parallel imaging on while the red stars represent parallel imaging off. Using saturation resulted in T_1 differences between parallel imaging on and off of on average 10%. When saturation was turned off, the mean T_1_s for the 5 different vials agreed.
**Figure S8:** (a) NLLS fit (dashed blue line) to the acquired data (red stars) with saturation on and the corresponding residual plot for one pixel (143,111) in the phantom. The sum of squared residuals (SSR) was 324.5. (b) NLLS fit (dashed blue line) to the acquired data (red stars) with saturation off and the corresponding residual plot with a SSR of 16.7.
**Figure S9:** Inversion‐recovery spin echo gold standard T1 map of the (a) T1phantom with mean T1s varying between 367 ms and 1699 ms, used for validation of the incomplete spoiling experiments and the (b) Calimetrix phantom used for validation of the spatial saturation experiments. Vials numbered 1 to 5 correspond to T1s of 548 ms, 633 ms, 790 ms, 1044 ms and 1429 ms, respectively. Vials above or below the numbered vials, with varying T2^*^ and fat‐fractions, respectively were not used in the analysis.

## Data Availability

The code for the 3D VFA SPGR $T_{1}$ mapping including the incomplete spoiling correction algorithm is available here: https://github.com/gabrielaBelsley/3D‐T1‐Mapping‐Siemens.
