# Supplementary material for: Incomplete Spoiling Correction for 3D T1 Mapping
Source: Magn Reson Med. 2026 Aug 12;96(6):2848–56. doi: 10.1002/mrm.70559 (PMC13619879; doi:10.1002/mrm.70559)
Supplement: Supplementary file 1 [file MRM-96-2848-s001.docx]

**Supporting Information**

**Incomplete Spoiling Correction**

The spoiled gradient recalled (SPGR) echo signal in the transverse plane was simulated using Extended-Phase-Graphs (EPG) for a range of parameters:

- $T_{1}$s varying between 500 ms and 1600 ms in steps of 100 ms;
- Excitation flip angles (FAs) varying between 1°-20° in steps of 1°.
- *T*2 of 30ms;
- TR of 4.1ms;
- RF phase increment of 50° (used in Siemens Prisma scanners).

The ratio between the theoretical steady-state SPGR signal and the EPG-simulated signal is the incomplete spoiling correction that transforms the acquired signal back to the steady-state domain. Correction curves were calculated across the grid of $T_{1}$and FA values, as illustrated in Figure S1. These curves were then interpolated based on initial $T_{1}$estimates and excitation FAs corrected for $B_{1}^{+}$ effects. The acquired SPGR signal was multiplied by the interpolated correction factor and fitted to the steady-state model to refine the initial $T_{1}$ estimate. The incomplete spoiling correction was iterated at each step with the updated $T_{1}$ estimate (Figure S2). Iterations stopped when consecutive T₁ estimates differed by <1 ms or after 5 iterations, as typically only 2 or 3 iterations were needed.

Figure S1. Incomplete spoiling correction factor given by the ratio between the theoretical steady-state signal and the extended-phase-graph signal as a function of the true FA in degrees for a range of $T_{1}s$varying between 500 ms (blue) and 1600 ms (green). Representative $T_{1}$values, observed in the liver of healthy volunteers, of 800 ms (orange), 1000 ms (yellow) and 1200 ms (purple) are also plotted. This plot shows the correction factor varies with the true FA as well as $T_{1}$, hence the iterative procedure for fitting $T_{1}$.

Figure S2 shows a flow diagram depicting the $T_{1}$ mapping algorithm developed with the incomplete spoiling correction highlighted in red.

Figure S2. Flow diagram to calculate the $T_{1}$ map corrected for incomplete spoiling using extended–phase-graph simulations and corrected for $B_{1}^{+}$ inhomogeneitffies using a $B_{1}^{+}$ map.

$\boldsymbol{B}_{\boldsymbol{1}}^{\boldsymbol{+}}$ **and** $\boldsymbol{B}_{\boldsymbol{0}}$**mapping acquisition parameters**

$B_{1}^{+}$ mapping used a 2D multi-slice GRE EPI sequence with fat saturation and nominal FAs of ${65}^{\circ}$ and ${130}^{\circ}$^18^. Each FA was a 10 s breath-hold. Acquisition parameters were: FoV=$450\times366$ $mm^{2}$, matrix=$64\times52$, slice thickness/spacing 8/2 mm, 15 slices interleaved, TR/TE=10000/11 ms without acceleration. A BW of 3906 Hz/pixel achieved a minimum echo spacing of 0.3 ms.

$B_{0}$ mapping used a 2D multi-slice double echo spoiled GRE acquisition with magnitude and phase reconstruction. Acquisition parameters were: were TR/TE=20/[4.78, 7.17] ms, FA=${15}^{\circ}$, FoV=$450\times380 mm^{2}$, matrix=$64\times54$, slice thickness/spacing 8/2 mm, 15 slices, monopolar readout gradients, BW=630 Hz/pixel, GRAPPA^21^ two-fold acceleration in the PE direction, acquisition time 8.6 s per breath-hold.

Figure S3 shows the liver mask in red drawn on the VFA SPGR high resolution image at FA 15° (high contrast). This mask was down sampled and applied to the $B_{0}$ and $B_{1}^{+}$ maps. Only the pixels in the $B_{1}^{+}$ map mask were used to calculate $T_{1}$, resulting in an automatic liver segmentation of the $T_{1}$ maps.

Figure S3. Liver mask in red drawn on the VFA SPGR high resolution image at FA 15°.

$\boldsymbol{T}_{\boldsymbol{1}}$**Fitting Accuracy and Residual Analysis Before and After Incomplete Spoiling Correction**

Without correcting the signal for incomplete spoiling, the fitted $T_{1}$was 1090ms and the NLLS fit resulted in a sum of squared residuals (SSR) of 43.0 (Figure S4), for one representative voxel in the phantom.

Figure S4. (a) $T_{1}$Map of the *FDA4-T1* phantom without correcting for incomplete spoiling. Red point showing the location of the voxel (126,176) used in the analysis of residuals. (b) NLLS fit (dashed blue line) to the acquired data (red stars) without correcting for incomplete spoiling and the corresponding (c) residuals. The fitted $T_{1}$was 1090 ms and the sum of the residuals squared (SSR) was 43.0 (arbitrary signal units). The residuals showed a pattern similar to the steady-state model curve indicating an underlying bias.

The residuals showed a pattern similar to the steady-state model curve indicating an underlying bias. After correcting the signal acquired for incomplete spoiling, the data fitted better to the steady state model and consequently the SSR decreased to 7.7 (Figure S5). The fitted $T_{1}$was 1016ms which is very close to the gold-standard $T_{1}$of 1022ms. For an SNR of 157 at a FA of 3°, the noise level was 2.2, and the residuals of the fit to the corrected data were all less than or equal to this noise level.

Figure S5. (a) $T_{1}$Map of the *FDA4-T1* phantom with incomplete spoiling correction. Red point showing the same voxel analysed in Figure S3. Note the change in colour for this vial corresponding to a lower $T_{1}$compared to Figure S3. (b) NLLS fit (dashed blue line) to the acquired data corrected for incomplete spoiling (red stars). The residuals (c) are smaller than in Figure S3 with a sum of squared residuals (SSR) equal to 7.7. The fitted $T_{1}$was 1016 ms, closer to the gold standard IR SE $T_{1}$of 1022 ms.

**Gradient Spoiling**

The 3D SPGR sequence uses a combination of gradient spoiling and RF spoiling with a RF phase increment of 50°. The extended phase graph simulations simulate this dephasing as this is the mechanism by which the transverse state shifts to a higher order state per TR. We note that gradient spoiling alone is insufficient even above 2π, because the higher-order magnetization is flipped to a negative state by subsequent RF pulses and can refocus, producing unwanted echoes — which is why RF spoiling is also required.

Calculating the phase distribution of isochromats per voxel for the product pulse sequence gives a total dephasing of 7.26 radians along the readout direction (slightly above 2$\pi$) plus a spoiler gradient along the slice direction of 3.7 radians, which further increases the total dephasing. This places the sequence in the weak spoiling regime as defined by Yarnykh^1^ and Corbin et al. ^2^, where gradient spoiling alone induces a 2π dephasing per voxel and diffusion effects are minor. This information was added to the supplementary information.

The signal was simulated using EPG with spoiler gradients ranging from 2π dephasing (weak spoiling regime) to 10π (strong spoiling regime) in steps of 2π/10, including diffusion effects using an apparent liver diffusion coefficient of 1.1×10^-3^ mm²/s (TR = 4.1 ms, flip angles 2°/2°/15°/15°, $T_{1}$= 800 ms, T2 = 30 ms), without applying the incomplete spoiling correction. Figure S6 shows that the $T_{1}$ error decreases as the spoiler gradient area increases, consistent with the findings of Yarnykh^1^ and Corbin et al. ^2^, where diffusion-enhanced dephasing in the presence of larger spoiler gradients reduces residual transverse magnetization. Thus, a smaller correction is needed. Nevertheless, as shown both in our simulations and in these references, larger spoiler gradients do not eliminate the need for post-hoc correction.

For our acquisition, with a short TR of 4.1 ms and small gradient spoiling, diffusion effects are negligible. The rms diffusion displacement during one TR is $\sqrt{\left\langle x^{2} \right\rangle}=\sqrt{2*1.1{*10}^{-3}\mathrm{mm}^{2}s^{-1}*4.1{*10}^{-3}s}=3\mu m$, considerably smaller than the voxel size (1.4 mm) which results in an irreversible phase spread due to diffusion alone of $\gamma A_{G}\sqrt{\left\langle x^{2} \right\rangle}$ =0.016 radians. For comparison, the smallest gradient area tested by Yarnykh^1^ (32.7 mT*ms/m, TR = 10 ms, D = 2.2×10⁻³ mm²/s) results in a rms diffusion displacement of ~6.6 μm per TR and an irreversible diffusion driven dephasing approximately four times larger than in our acquisition. Using large spoiler gradients (≥280 mTms/m as recommended by Yarnykh) would require a substantially longer TR to accommodate the gradient waveform, which would make breath-hold liver scanning impossible. Furthermore, such gradients would introduce meaningful diffusion sensitivity, requiring measurement of diffusion coefficient as it varies between healthy and cirrhotic liver.

Figure S6. Error in $T_{1}$ for SPGR EPG simulations with varying spoiler gradient areas corresponding to dephasing of 2pi (33.55 mT/m), increasing in steps of 2pi/10 until 10pi (167.75 mT/m) and including diffusion (blue) with diffusion coefficient of 1.1×10^-3^ mm²/s and excluding diffusion effects (red). TR=4.1 ms, $T_{1}$=800ms, T2=30 ms, spoiler gradient duration =0.5 ms, pixel size 1.4 mm.

**Spatial Saturation**

For the SPGR pulse sequence, there were two saturation pulses at the start of the block followed by 36 slab selective imaging excitation pulses of approximately 0*.*7 ms duration. The time interval between adjacent imaging pulses was equal to one TR of duration 4*.*1 ms. The train of 36 RF pulses, representing 36 partitions, lasted approximately 148 ms before the next set of two saturation pulses were applied. The two saturation pulses introduce a gap of 18.6 ms that is much larger than TR. During this time the longitudinal magnetisation growth is larger than the amount of magnetisation flipped by the 3D SPGR excitation pulse, destroying the steady state signal.

Figure S7 (a) shows differences in $T_{1}$between parallel imaging on and off with saturation turned on, which is not expected as parallel imaging should not change the SPGR signal. With saturation off, the $T_{1}$values agreed independent of whether acceleration was used (Figure S6 (b)). Parallel imaging should not affect the $T_{1}$estimate. However, the use of acceleration along the partition direction will result in less partitions between each set of saturation pulses and result in a different perturbation of the steady-state signal compared to not using acceleration.

Figure S7. $T_{1}$error in percent, relative to the gold standard method inversion recovery spin echo, with saturation (a) on and (b) off. Blue stars represent parallel imaging on while the red stars represent parallel imaging off. Using saturation resulted in $T_{1}$differences between parallel imaging on and off of on average 10%. When saturation was turned off, the mean $T_{1}$s for the 5 different vials agreed.

A further indication that saturation affects the $T_{1}$estimation are the increased residuals observed in the fit when saturation was turned on (Figure S8).

Figure S8. (a) NLLS fit (dashed blue line) to the acquired data (red stars) with saturation on and the corresponding residual plot for one pixel (143,111) in the phantom. The sum of squared residuals (SSR) was 324.5. (b) NLLS fit (dashed blue line) to the acquired data (red stars) with saturation off and the corresponding residual plot with a SSR of 16.7.

**Gold Standard** $\boldsymbol{T}_{\boldsymbol{1}}$ **Maps**

A gold standard (GS) $T_{1}$ map using a slice-selective inversion-recovery spin echo (IR SE) was acquired on the $T_{1}$phantom using TIs of [25, 50, 75, 100, 200, 300, 400, 600, 800, 1200, 1600, 2300, 3000, 4000, 5000]ms, TR/TE=9s/12ms, FoV=$225\times300 mm^{2}$, matrix=$144\times192$, 6mm slice thickness, BW=130 Hz/pixel, Phase Partial Fourier Off, Interpolation On, no acceleration. The acquisition time for each TI was 21 minutes and 47 seconds.

To calculate the GS $T_{1}$ map (Figure S9), the signal in each pixel was fit using a three-parameter non-linear IR model:

$$S_{n}=a-be^{\left( \frac{-TI_{n}}{T_{1}} \right)} ,$$

where $S_{n}$ is the signal intensity, $a$ is the signal at $TI=\infty$, after its full recovery to the equilibrium longitudinal magnetisation $M_{z}$, and $a-b$ is the signal at $TI=0$. Initial guesses for $a$ is the signal from the largest TI (5000 ms) and for $b$ is two times the signal at the largest TI. $T_{1}$ was initialised to 500 ms. The subscript $n$ denotes the index of the TI; in this work the signal was measured at 15 different TIs. For each fit, the TI corresponding to the minimum signal intensity is determined and all the TIs less than or equal to the TI with the minimum signal intensity are assigned a negative value, i.e., samples which have not reached the null intensity. A second fit is carried out with the minimum signal intensity changed back to positive. The fit with the smallest sum squared errors value is chosen.

Figure S9. Inversion-recovery spin echo gold standard $T_{1}$ map of the (a) $T_{1}$phantom with mean $T_{1}$s varying between 367 ms and 1699 ms, used for validation of the incomplete spoiling experiments and the (b) Calimetrix phantom used for validation of the spatial saturation experiments. Vials numbered 1 to 5 correspond to $T_{1}$s of 548 ms, 633 ms, 790 ms, 1044 ms and 1429 ms, respectively. Vials above or below the numbered vials, with varying *T*2* and fat-fractions, respectively were not used in the analysis.
